# The impact of multimorbidity on adult physical and mental health in low- and middle-income countries: what does the study on global ageing and adult health (SAGE) reveal?

**DOI:** 10.1186/s12916-015-0402-8

**Published:** 2015-08-03

**Authors:** Perianayagam Arokiasamy, Uttamacharya Uttamacharya, Kshipra Jain, Richard Berko Biritwum, Alfred Edwin Yawson, Fan Wu, Yanfei Guo, Tamara Maximova, Betty Manrique Espinoza, Aarón Salinas Rodríguez, Sara Afshar, Sanghamitra Pati, Gillian Ice, Sube Banerjee, Melissa A. Liebert, James Josh Snodgrass, Nirmala Naidoo, Somnath Chatterji, Paul Kowal

**Affiliations:** International Institute for Population Sciences, Mumbai, India; Department of Community Health, University of Ghana, Accra, Ghana; Shanghai Municipal Center for Disease Control and Prevention (CDC), Shanghai, China; Russian Academy of Medical Sciences (RAMS), Moscow, Russian Federation; National Institute of Public Health (INSP), Centre for Evaluation Research and Surveys, Cuernavaca, Morelos Mexico; Academic Unit of Primary Care and Population Sciences, Faculty of Medicine, University of Southampton, University Road, Southampton, SO17 1BJ UK; Indian Institute of Public Health, Bhubaneswar, Public Health Foundation of India, Bhubaneswar, Odisha India; Ohio University, Department of Social Medicine and Director of Global Health, Athens, OH USA; Centre for Dementia Studies, Brighton and Sussex Medical School, University of Sussex, Brighton, UK; University of Oregon, Department of Anthropology, Eugene, OR USA; World Health Organization, Statistics Measurement and Analysis Unit, Geneva, Switzerland; World Health Organization Study on global AGEing and adult health (SAGE), Geneva, Switzerland; University of Newcastle Priority Research Centre for Gender, Health and Ageing, Newcastle, NSW Australia

**Keywords:** Activities of daily living, Low- and middle-income countries, Mental health, Multimorbidity, Non-communicable diseases, Quality of life

## Abstract

**Background:**

Chronic diseases contribute a large share of disease burden in low- and middle-income countries (LMICs). Chronic diseases have a tendency to occur simultaneously and where there are two or more such conditions, this is termed as ‘multimorbidity’. Multimorbidity is associated with adverse health outcomes, but limited research has been undertaken in LMICs. Therefore, this study examines the prevalence and correlates of multimorbidity as well as the associations between multimorbidity and self-rated health, activities of daily living (ADLs), quality of life, and depression across six LMICs.

**Methods:**

Data was obtained from the WHO’s Study on global AGEing and adult health (SAGE) Wave-1 (2007/10). This was a cross-sectional population based survey performed in LMICs, namely China, Ghana, India, Mexico, Russia, and South Africa, including 42,236 adults aged 18 years and older. Multimorbidity was measured as the simultaneous presence of two or more of eight chronic conditions including angina pectoris, arthritis, asthma, chronic lung disease, diabetes mellitus, hypertension, stroke, and vision impairment. Associations with four health outcomes were examined, namely ADL limitation, self-rated health, depression, and a quality of life index. Random-intercept multilevel regression models were used on pooled data from the six countries.

**Results:**

The prevalence of morbidity and multimorbidity was 54.2 % and 21.9 %, respectively, in the pooled sample of six countries. Russia had the highest prevalence of multimorbidity (34.7 %) whereas China had the lowest (20.3 %). The likelihood of multimorbidity was higher in older age groups and was lower in those with higher socioeconomic status. In the pooled sample, the prevalence of 1+ ADL limitation was 14 %, depression 5.7 %, self-rated poor health 11.6 %, and mean quality of life score was 54.4. Substantial cross-country variations were seen in the four health outcome measures. The prevalence of 1+ ADL limitation, poor self-rated health, and depression increased whereas quality of life declined markedly with an increase in number of diseases.

**Conclusions:**

Findings highlight the challenge of multimorbidity in LMICs, particularly among the lower socioeconomic groups, and the pressing need for reorientation of health care resources considering the distribution of multimorbidity and its adverse effect on health outcomes.

**Electronic supplementary material:**

The online version of this article (doi:10.1186/s12916-015-0402-8) contains supplementary material, which is available to authorized users.

## Background

Low- and middle-income countries (LMICs) in the 21st century are witnessing an unprecedented upward shift in life expectancy [1]. This is causing changes in the disease burden profiles of LMICs, with chronic non-communicable diseases (NCDs) becoming more common and growing public health challenge [2–5]. As a result of increasing longevity, multiple comorbid conditions, commonly referred to as ‘multimorbidity’, have also become progressively more common among older adults [6–8]. Evidence from both high- and low-income countries indicates that older adults are at much higher risk for multiple chronic diseases [9–14]. At the same time, several recent studies have provided evidence that younger adults also have substantial prevalence of multimorbidity [15–19].

Socioeconomic status (SES) has been found to be strongly associated with the prevalence of multimorbidity, regardless of whether SES is measured through education [20, 21], income [22], occupation [23], or area-based deprivation [24]. In a review of 26 studies on multimorbidity focused on East-Mediterranean countries, Boutayab et al. [25] show that low income, low level of education, and unemployment are associated with higher prevalence of multimorbidity.

Multimorbidity has been associated with adverse health outcomes, such as reduced physical function [26, 27], poor quality of life [28], poor self-rated health (SRH) [29, 30], increased use of inpatient and ambulatory care [13, 31], and mortality [10]. More than two decades ago, Verbrugge et al. [32] demonstrated the exponential increase in disability with increasing numbers of chronic diseases. Multimorbidity also raises the complexities of clinical treatment and patient management, and is consequently associated with higher medical care costs owing to the need for long-term care [17, 33]. The adverse impact of multimorbidity on other health domains is further exacerbated by socioeconomic deprivation and poorly prepared medical care facilities [34–36].

Studies investigating the prevalence and determinants of multimorbidity have primarily focused on high-income countries (HICs). Multimorbidity studies in LMICs are limited in geographical scope, the number of diseases studied and the effects of multimorbidity (absolute number of diseases or disease combinations) on health outcomes such as physical functioning, quality of life, or mental health [12, 22, 25, 37, 38]. Of the few existing studies on multimorbidity prevalence from LMICs, Khanam et al. [12] estimated the prevalence of multimorbidity to be 53.8 % among the older adult population of Bangladesh. Alaba and Chola [22] analyzed the adult (aged 18+) population of South Africa and reported 4 % to have multimorbidity, with over 70 % of these adults being women. The large and growing burden of NCDs in LMICs, particularly when coupled with limitations in resources and competing public health priorities points, to the need to understand the burden of multimorbidity [39, 40].

In the present study, data from the World Health Organization’s multi-country Study on global AGEing and adult health (WHO SAGE) Wave 1 was used to investigate two main objectives: 1) to explore the prevalence and SES correlates of multimorbidity in adults, and 2) to examine the associations between multimorbidity and four main health outcomes: self-rated overall general health, depression, physical functioning, and subjective well-being.

### Hypotheses

This study aims to test the following hypotheses: 1) LMICs will exhibit a negative association between higher SES and multimorbidity, similar to the evidence from HICs and some developing countries, and 2) multimorbidity will have positive associations with other health-related outcomes, namely lower SRH, depression, limitation in activities of daily living (ADLs), and poorer quality of life.

### Data sources

This study used data from SAGE Wave 1 (2007–2010). SAGE is a longitudinal ageing and health study with nationally representative samples of adults from six countries: China, Ghana, India, Mexico, the Russian Federation, and South Africa. These countries are at different stages of the demographic and epidemiological transitions but are (with the exception of Russia) experiencing a rapid rise in the older adult population [41]. SAGE is designed as a multi-wave panel study representative of the population aged 50 and older, with a smaller cohort of respondents aged 18–49 for comparative purposes. All sampling plans use multistage clustered design samples drawn from an updated frame. Each household and individual is assigned a known non-zero probability of being selected [42]. Household and individual weights were post-stratified to weight up to population distributions by age and sex in each country. Detailed description of study and sample design is provided elsewhere [42, 43].

## Methods

### Chronic conditions and multimorbidity

Multimorbidity is defined as the simultaneous presence of two or more chronic physical health conditions. For this analysis, eight chronic health conditions were included, namely angina pectoris, arthritis, asthma, chronic lung disease, diabetes mellitus, hypertension, stroke, and low visual acuity.

Of these eight conditions, diabetes mellitus and stroke were assessed through a question about ever being diagnosed with the disease by a health professional. The specific question was, “Have you ever been told by a health professional/doctor that you have (disease name)?”

The prevalence of angina pectoris, arthritis, asthma, and chronic lung disease was derived from a set of symptom-based questions, combined with a diagnostic algorithm. The symptomatic questions and algorithm for each of the diseases are presented in the supplementary material (Additional file 1). Additionally, the use of treatment/medication received in the 12 months prior to interview was indicative of a diagnosis and was included in prevalence estimates for each disease. Prevalence of angina, arthritis, and asthma was based on symptom-reporting and diagnostic algorithm, adjusted for treatment/medication received in the 12 months prior to interview.

The assessment of hypertension and visual acuity was based on direct physical examination undertaken at the time of interview. The prevalence of hypertension was based on measured blood pressure (systolic and diastolic) taken with the respondent in a seated position. An average of the second and third of three total readings was used as the outcome. In accordance with WHO/ISH guidelines for the management of hypertension [44], the limit for high systolic blood pressure was 140 mm/hg or above, and for diastolic blood pressure 90 mm/hg or above. An individual was considered to be hypertensive if average systolic or diastolic blood pressure readings exceeded either of these thresholds or they reported current treatment for hypertension.

Visual acuity was measured for both near and distance vision in each eye using a tumbling “E” logMAR chart [45]. Measured near and distance visual acuity was classified into normal vision (0.32–1.6 decimal) and low vision (0.01–0.25 decimal) [46]. In this study, a respondent had low vision if they had either low near or distance vision in both eyes.

### Health outcomes

#### Self-rated health (SRH)

The specific question used to assess overall general SRH was, “In general, how would you rate your health today?” A five-point response scale was used: very good, good, moderate, bad, and very bad. For this analysis, bad and very bad health responses were combined as ‘poor health’ and remaining categories combined into ‘good health’ to generate a dichotomous health variable. Poor SRH is the outcome used in the analysis.

#### Physical functioning: activities of daily living (ADL)

Limitation in ADL was used to assess physical functioning. The questions were based on self- reported difficulty in engaging in activities during the last 30 days, using a five-point response scale ranging from none to extreme difficulty. The ADL measure included in SAGE was based on WHODAS 2.0 and has been validated in LMICs by WHO and collaborating agencies [47]. WHODAS 2.0 is validated cross-culturally through a systematic research study. The cross-cultural applicability research study used various qualitative methods to explore the nature and practice of health status assessment in different cultures. The study included linguistic analysis of health-related terminology, key informant interviews, focus groups, and quasi-quantitative methods such as pile sorting and concept mapping (carried out in tandem). Information was gathered on the conceptualization of disability and on important areas of day-to-day functioning.

In this study, severe and extreme difficulties were combined to represent limitation in a particular activity. We have used an extended set of ADL that included sitting for long periods, walking 100 m, standing up, standing for long periods, climbing one flight of stairs, stooping/kneeling/crouching, picking up things with fingers, extending arms above shoulders, concentrating for 10 min, walking a long distance (1 km), bathing, getting dressed, carrying things, moving around inside home, getting up from lying down, and getting to and using the toilet. For the analysis, a dichotomous variable was created, which took value 1 if the respondent noted a limitation in one or more of the above ADLs (1+ ADL) and 0 otherwise.

#### Quality of life

The 8-item WHO Quality of Life (WHOQoL) instrument was used to quantify quality of life and included two questions in each of four broad domains: physical, psychological, social, and environmental [48]. Quality of life was assessed by asking respondents to rate their satisfaction with different domains of their lives, such as with money, health, and relationships, as well as rating their overall life satisfaction, using a five-point response scale, ranging from very satisfied to very dissatisfied. A composite score was created by summing the responses across the different questions and rescaling the result from 0–100 where a higher score indicated better quality of life.

#### Mental health

Depression was used as a measure of mental health. Depression was assessed through a set of symptomatic questions based on the World Mental Health Survey version of the Composite International Diagnostic Interview [49]. Diagnosis of major depressive episode was derived from an algorithm that accounted for reporting symptoms of depression during the past 12 months [50]. The detailed symptomatic questions and algorithm are provided in the supplementary material (Additional file 1). Prevalence was based on the result of the diagnostic algorithm, adjusting for treatment received.

### Indicators of socioeconomic status and control variables

Years of schooling and household wealth quintile were used to represent SES. For analytical convenience, the highest number of years of education completed was grouped into four categories: no formal schooling, 1–5 years, 6–9 years, and 10 or more years of schooling. In addition to education, household wealth was used as an alternate measure of SES. A wealth index was derived from the household ownership of durable goods, dwelling characteristics (type of floors, walls and cooking stove), and access to services such as improved water, sanitation, and cooking fuel. The detailed list of items is given in the supplementary material (Additional file 1). The results were recoded into dichotomous variables taking the value of 0 if the household did not possess or have access to the good or service, and 1 if it did. A pure random effect model was used to estimate assets per household, then an ‘asset ladder’ was generated for each country [51]. Using a Bayesian post-estimation (empirical Bayes) method, households were arranged on the asset ladder, where the raw continuous income estimates were transformed in the final step into quintiles.

Two sets of control variables, demographic factors and health risk factors, were included in this study. The demographic variables included: age groups (18–49, 50–59, 60–69, 70+), locality (urban or rural), sex (men or women), and marital status (currently married/cohabiting or all other). The health risk variables consisted of tobacco use [current users (daily or non-daily) or non-user]; alcohol consumption [current user (consumed 1–4 days/week in the last 12 months) or non-user]; physical activity (active [involved in 150+ minutes of vigorous activity or 300+ minutes of moderate activity per week] or otherwise inactive); high risk waist-to-hip ratio (cutoff point: ≥0.90 for men and ≥0.85 for women); and obesity classification (BMI ≥30).

### Statistical methods

A two-stage statistical analysis was undertaken; first, the correlates of any morbidity and multimorbidity (2+ chronic diseases) were examined using a multinomial logit model. Second, the association between multimorbidity and the four primary health outcomes were examined: 1+ ADL, presence of depression, poor SRH, and low WHOQoL score. Of the four health outcomes, three were binary variables, 1+ ADL limitation, depression, and poor SRH; therefore, logit models were used to examine the association of morbidity with these indicators. Linear regression was used to investigate associations with the WHOQoL index. All regressions were run on the pooled data from the six countries and therefore estimated in a multilevel framework. Random intercept multilevel (three-level) models were used, where country was the highest level, state/province of residence the second level, and individuals the first level. All analyses were carried out in STATA 12.0. The estimates were considered significant if *P* <0.10.

### Ethical approval

SAGE was approved by the World Health Organization’s Ethical Review Committee. Additionally, partner organizations in each country implementing SAGE obtained ethical clearance through their respective institutional review bodies.

### Informed consent

Written informed consent was obtained from all study participants.

## Results

### Sample characteristics

This study analyzed data from 42,236 adults (18,243 men and 23,993 women) aged ≥18 years from WHO SAGE Wave 1 in China, Ghana, India, Mexico, Russia, and South Africa. Table 1 presents the percentage distribution of socioeconomic and demographic characteristics of the study population from each country. Among the countries, the Russian Federation had the highest percentage of population aged 70 years and above (12.5 %) compared to less than 10 % for the other countries. The proportion living in rural areas ranged from 19 % in Russia to 75 % in India. The percentage of population with no formal schooling was highest in India (36.2 %) followed by Ghana (32.7 %) and lowest for Russia (0.3 %). The percentage of population with 10+ years of schooling was highest in Russia (87 %) compared to less than 30 % for Ghana (29.4 %), India (26.8 %), and Mexico (25.7 %).

**Table 1:** Percent distribution of selected socio-demographic characteristics, by country and for the pooled sample, WHO SAGE Wave 1 (2007/10)

	China	Ghana	India	Mexico	Russia	South Africa	All countries, total (pooled)	Men (pooled)	Women (pooled)
N	14,793	5108	11,230	2732	4152	4221	42,236	18,243	23,993
Age Group									
18–49	74.2	75.4	75.2	73.6	58.7	75.9	70.7	71.8	69.5
50–59	11.6	9.8	12.0	12.7	18.7	12.0	14.6	14.5	14.7
60–69	8.2	6.8	7.7	6.8	10.2	7.4	8.4	8.2	8.6
70+	6.0	8.1	5.1	7.0	12.5	4.7	6.4	5.6	7.2
Sex									
Male	50.9	50.0	50.9	48.0	45.0	47.2	50.7	–	–
Female	49.1	50.0	49.1	52.0	55.0	52.8	49.3	–	–
Residence									
Urban	48.5	45.8	25.5	77.8	81.5	69.3	44.6	44.0	45.2
Rural	51.5	54.2	74.5	22.2	18.5	30.7	55.4	56.0	54.8
Marital status									
Never married	5.7	8.4	9.4	21.3	12.8	31	8.4	10.5	6.3
Currently married/Cohabiting	89.0	72.6	81.9	69.6	61.1	52.8	83.3	85.3	81.3
Widowed/Divorced/Separated	5.3	19.0	8.7	9.1	26.0	16.1	8.3	4.2	12.4
Years of schooling									
No formal schooling	8.4	32.7	36.2	5.9	0.3	7.8	18.3	11.3	25.6
1–5 years	16.0	9.6	17.6	20.2	2.4	15.1	16.2	15.6	16.9
6–9 years	46.2	18.9	19.4	48.3	10.3	26.9	33.5	35.6	31.5
10+ years	29.4	38.8	26.8	25.7	87.0	50.2	31.9	37.6	26.1
Wealth quintile									
Lowest	9.8	15.3	20.6	16.5	12.7	18.9	14.3	14.5	14.1
Second	15.9	17.9	21.2	23.3	12.8	19.5	17.9	17.7	18.0
Middle	18.3	19.1	19.9	20.1	16.5	20.5	19.0	20.1	17.9
Fourth	23.4	22.6	18.0	15.4	23.5	19.4	21.3	20.7	21.8
Highest	32.6	25.2	20.2	24.6	34.5	21.8	27.6	27.0	28.2

Results are weighted using pooled sampling weights

### Prevalence of multimorbidity and the primary health outcome measures

Table 2 shows the prevalence of having any single chronic disease, multimorbidity (2+ chronic diseases), 1+ ADL limitation, depression, poor SRH, and low mean WHOQoL index based on the pooled sample of the six SAGE countries. Overall, the prevalence of morbidity – defined as the presence of at least one of the eight chronic diseases – was 54.2 %. The prevalence of having at least one chronic disease was highest in South Africa (69.4 %) followed by Ghana (62.1 %), and lowest in India (51.6 %). The overall prevalence of multimorbidity was 21.9 %. Among the six countries, Russia had the highest prevalence of multimorbidity (34.7 %) and the lowest was observed in China (20.3 %); the remaining four countries had a multimorbidity prevalence of approximately 22 %.

**Table 2:** Prevalence of any morbidity, multimorbidity, and four health outcome measures by background characteristics, WHO-SAGE Wave 1 (2007/10)

Background variables	Any morbidity, % (95 % CI)	Multimorbidity, % (95 % CI)	1+ ADL, % (95 % CI)	Depression, % (95 % CI)	Poor self-rated health, % (95 % CI)	Mean WHOQoL score (95 % CI)
Age group						
18–49	43.8 (41.9–45.7)	12.3 (11.3–13.5)	9.1 (8.2–10.0)	4.9 (4.2–5.7)	7.4 (6.5–8.5)	55.4 (54.9–55.9)
50–59	73.5 (71.7–75.2)	34.8 (33.1–36.4)	16.8 (15.5–18.3)	6.4 (5.1–7.9)	16.2 (15.0–17.5)	53.3 (52.8–53.8)
60–69	83.5 (82.1–84.8)	50.1 (48.5–51.7)	27.7 (25.9–29.6)	8.2 (7.2–9.3)	22.3 (20.6–24.1)	51.5 (50.8–52.3)
70+	87.7 (85.8–89.4)	60.7 (58.4–62.9)	44 (41.7–46.4)	9.6 (8.4–10.9)	33.6 (31.6–35.6)	49.2 (48.4–50.0)
Sex						
Male	53.3 (51.1–55.5)	19.0 (17.7–20.3)	10.1 (9.1–11.2)	4.7 (4.0–5.6)	10.1 (9.1–11.3)	55.1 (54.6–55.7)
Female	55.2 (53.3–57.0)	24.8 (23.5–26.2)	18 (16.8–19.2)	6.7 (5.9–7.5)	13.1 (12.2–14.1)	53.7 (53.1–54.2)
Residence						
Urban	51.3 (48.7–54.0)	20.5 (18.8–22.4)	10.0 (8.7–11.4)	5.0 (3.9–6.4)	8.4 (7.5–9.4)	55.5 (54.7–56.2)
Rural	56.6 (54.8–58.4)	22.9 (21.9–24.1)	17.2 (16.2–18.3)	6.2 (5.6–7.0)	14.2 (13.1–15.3)	53.5 (53.0–54.1)
Years of schooling						
No schooling	66.0 (63.7–68.2)	35.2 (33.2–37.3)	33.6 (31.4–35.9)	12.0 (10.6–13.5)	18.6 (17.2–20.1)	51.0 (50.1–51.9)
1–5 years	63.0 (60.3–65.6)	28.0 (25.6–30.6)	18.7 (16.5–21.1)	5.8 (4.7–7.2)	18.6 (16.2–21.2)	51.7 (50.9–52.5)
6–9 years	52.3 (49.8–54.8)	17.5 (16.0–19.0)	8.7 (7.6–9.9)	4.3 (3.5–5.4)	10.6 (9.2–12.2)	54.3 (53.7–54.9)
10+ years	45.3 (42.3–48.4)	16.0 (14.0–18.3)	6.3 (5.5–7.2)	3.7 (2.9–4.6)	5.2 (4.4–6.2)	57.5 (56.8–58.3)
Wealth quintile						
Lowest	59.4 (56.7–62.0)	28.2 (25.9–30.7)	24.2 (21.7–26.9)	8.5 (7.0–10.2)	19.4 (17.3–21.6)	48.0 (47.2–48.8)
Second	57 (53.9–60.1)	23.6 (21.6–25.6)	18.5 (16.5–20.6)	6.7 (5.6–8.0)	15 (13.3–16.8)	52.1 (51.4–52.8)
Middle	55.7 (51.8–59.6)	22.0 (19.9–24.3)	14.3 (12.8–16.0)	6.5 (5.4–7.8)	13.9 (12.3–15.6)	53.7 (53.0–54.5)
Fourth	56.9 (53.4–60.4)	21.9 (19.9–24.0)	11.1 (9.8–12.6)	5.2 (4.0–6.7)	10.2 (8.7–12.0)	55.6 (54.8–56.4)
Highest	46.7 (43.8–49.6)	17.3 (15.3–19.4)	7.6 (6.6–8.7)	3.5 (2.8–4.3)	4.9 (4.0–6.0)	58.7 (58.0–59.4)
Country						
China	55 (52.5–57.4)	20.3 (19.1–21.7)	5.6 (4.9–6.2)	1.6 (1.2–2.2)	12.5 (11.4–13.6)	54.9 (54.2–55.6)
Ghana	62.1 (58.6–65.4)	22.0 (19.3–25.1)	16.7 (14.7–18.9)	5.2 (4.0–6.7)	8.9 (7.4–10.8)	52.3 (51.4–53.1)
India	51.6 (49.8–53.4)	22.0 (20.5–23.5)	26.2 (24.6–27.9)	11.6 (10.1–13.2)	10.9 (9.8–12.1)	53.7 (53.1–54.4)
Mexico	52.7 (45.2–60.0)	22.1 (18.2–26.4)	19.0 (14.6–24.4)	9.3 (6.5–13.1)	6.9 (4.6–10.2)	55.3 (53.8–56.9)
Russia	59.7 (51.6–67.3)	34.7 (27.4–42.8)	11.6 (9.4–14.1)	4.9 (3.6–6.7)	11.3 (8.6–14.7)	53.5 (52.2–54.9)
South Africa	69.4 (62.2–75.8)	22.5 (18.2–27.4)	19.4 (14.9–24.9)	5.0 (2.5–9.9)	9.7 (7.0–13.3)	53.8 (51.7–55.8)
Number of diseases						
0	–	–	7.1 (6.2–8.1)	3.2 (2.5–4.0)	5.8 (4.9–7.0)	57.0 (56.3–57.6)
1	–	–	11.6 (10.6–12.6)	4.7 (3.9–5.7)	10.1 (8.9–11.4)	54.3 (53.7–54.9)
2	–	–	25.0 (22.6–27.4)	9.8 (8.2–11.6)	18.7 (16.8–20.7)	51.0 (50.2–51.8)
3	–	–	37.1 (33.5–40.8)	12.3 (10.1–14.9)	33.5 (30.2–36.9)	47.0 (45.9–48.0)
4+	–	–	58.7 (53.4–63.8)	27.0 (22.1–32.4)	50.0 (45.2–54.7)	43.3 (42.0–44.6)
Total	54.2 (52.7–55.8)	21.9 (20.9–22.9)	14.0 (13.2–14.8)	5.7 (5.0–6.4)	11.6 (10.9–12.4)	54.4 (53.9–54.9)

Percentage estimates are weighted by the pooled country weight. Higher WHOQoL score indicates better quality of life (on a scale of 0 to 100)

The prevalence of any single condition and of multimorbidity increased with each progressively older age group; 87 % of the population in the oldest age group (70+) had at least one chronic disease and 60.7 % had multimorbidity. Similarly, the prevalence of single morbidity and multimorbidity was consistently higher in those with lower levels of education and wealth, and lower in those with higher education and wealth (Table 2). For example, prevalence of multimorbidity was 35.2 % and any one morbidity was 66.0 % among adults with no formal schooling compared to 16.0 % and 45.3 % prevalence among adults with 10+ years of schooling.

Overall, 14 % of respondents had 1+ ADL limitation, 5.7 % had depression, 11.6 % reported poor SRH, and the mean WHOQoL score was 54.4 (on the scale of 0 to 100). Poorer health outcomes were generally more prevalent at older ages, in women, in rural dwellers, at lower SES, and with larger numbers of comorbid conditions. While considerable cross-country variations were observed in the prevalence of 1+ ADL, depression, poor SRH, and low mean WHOQoL scores, the overall patterns were consistent.

Table 2 also shows the prevalence of the four health outcomes among adults with different numbers of chronic conditions. With increasing numbers of chronic conditions, each of the four health outcome measures became worse. For instance, the percentage of adults with 1+ ADL increased eight-fold (from 7.1 % to 58.7 %), depression prevalence increased by nine-fold (3.2 % to 27 %), poor SRH increased six-fold (from 5.8 % to 50 %), and the mean WHOQoL score declined from 57.0 to 43.3 in those with no chronic diseases compared to those with four or more conditions, respectively.

### Associations between SES and multimorbidity

Both measures of SES were negatively associated with 1+ ADL, depression, and poor SRH, while positively associated with WHOQoL score (Table 2). For example, 33.6 % of respondents with no formal education had 1+ ADL compared to 6.3 % in those with 10+ years of schooling. The prevalence of depression was 12 % in the no formal schooling group versus 3.7 % in 10+ years schooling group, while poor SRH was 18.6 % in the no formal schooling group versus 5.2 % in the 10+ years of schooling group. Mean WHOQoL index score was worse (51.0) for respondents with no formal schooling compared to 57.5 for those with 10+ years of schooling. A similar pattern was observed for household wealth quintiles.

**Fig. 1:**
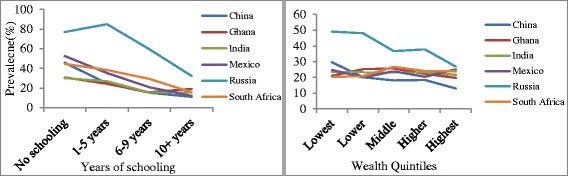
Prevalence of multimorbidity, by socioeconomic status measures and country, WHO SAGE Wave 1 (2007/10)

Figure 1 presents the prevalence of multimorbidity by years of schooling completed and household wealth quintiles, and by country. Years of schooling completed showed a pronounced negative association with the prevalence of multimorbidity for all countries, with the largest differences observed in Mexico and Russia. Small differences were seen in the prevalence of multimorbidity across wealth quintiles in all countries except Russia.

### Correlates of chronic disease and multimorbidity

Table 3 presents the estimates of multilevel multinomial logistic regression models used to examine the association of socioeconomic, demographic, and health risk factors with multimorbidity. The dependent variable had three categories: no disease, one disease, and 2+ diseases (multimorbidity). The ‘no disease’ category was considered as the reference group in the multinomial logit regression model. Table 3 shows that the relative risks of both one disease and multimorbidity (2+ diseases) increased at each higher age group compared to no disease. For example, compared to the 18–49 age group, adults in the 70+ age group were four times (RRR = 4.04) more likely to have one disease, and almost 18 times (RRR = 17.9) more likely to have multimorbidity relative to no disease. Compared with men, women were significantly more likely (RRR = 1.26) to have multimorbidity than no disease. Adults living in rural areas were less likely than urban dwellers (RRR = 0.95) to have multimorbidity relative to no disease. Measures of SES, based on years of schooling and wealth quintiles, were negatively associated with both multimorbidity and having one disease. Furthermore, all health risk factors, except tobacco consumption, were significantly associated with higher prevalence of both one disease and multimorbidity.

**Table 3:** Multilevel multinomial logit model estimates examining the correlates of multimorbidity

Variable	One disease versus no disease (95 % CI)	Multimorbidity versus no disease (95 % CI)
Age		
18–49 ^R^		
50–59	2.50*** (2.31–2.7)	5.10*** (4.68–5.58)
60–69	3.37*** (3.09–3.71)	10.75*** (9.73–11.85)
70+	4.04*** (3.58–4.52)	17.96*** (15.90–20.22)
Sex		
Male ^R^		
Female	1.04 (0.98–1.12)	1.26*** (1.17–1.35)
Residence		
Urban ^R^		
Rural	1.04 (0.97–1.11)	0.95* (0.88–1.01)
Marital status		
Never married ^R^		
Currently married/cohabiting	1.53*** (1.37–1.72)	1.59*** (1.41–1.77)
Widowed/divorced	1.74*** (1.54–2.03)	1.95*** (1.71–2.18)
Years of schooling		
No formal schooling ^R^		
1–5 years	0.96 (0.87–1.05)	0.92** (0.83–1.00)
6–9 years	0.79*** (0.72–0.86)	0.71*** (0.64–0.77)
10+ years	0.71*** (0.64–0.77)	0.53*** (0.48–0.59)
Wealth quintile		
Lowest ^R^		
Lower	1.06 (0.95–1.16)	1.02 (0.91–1.12)
Middle	1.12** (1.01–1.22)	1.04 (0.94–1.14)
Higher	1.03 (0.94–1.13)	0.92* (0.83–1.02)
Highest	1.02 (0.92–1.13)	0.87*** (0.77–0.96)
Waist-hip ratio		
High risk	1.12*** (1.05–1.19)	1.34*** (1.26–1.43)
Low risk ^R^		
Body mass index		
Obese	1.58*** (1.39–1.76)	2.26*** (2–2.52)
Not obese ^R^		
Physical activity		
Active ^R^		
Inactive	1.02 (0.95–1.1)	1.14*** (1.07–1.23)
Daily tobacco consumption		
No ^R^		
Yes	1.00 (0.93–1.08)	1.00 (0.93–1.09)
Alcohol consumption		
No ^R^		
Yes	1.14*** (1.04–1.27)	1.12** (1.01–1.24)
Random part		
Country level variance		
Variance (cons_1)	0.08 (0.02–0.27)	
Covariance (cons_1,cons_2)	0.04 (−0.09 to 0.28)	
Variance (cons_2)	0.25 (0.06–0.85)	
Province level variance		
Variance (cons_1)	0.12 (0.07–0.19)	
Covariance (cons_1,cons_2)	0.19 (0.12–0.3)	
Variance (cons_2)	0.36 (0.24–0.54)	

^R^ Reference category. * *P* <0.1, ** *P* <0.05, *** *P* <0.01. Estimates are obtained through MCMC algorithm available in MLWin

### ADL limitations, poor self-rated health (SRH), depression, and quality of life by number of diseases

**Fig. 2:**
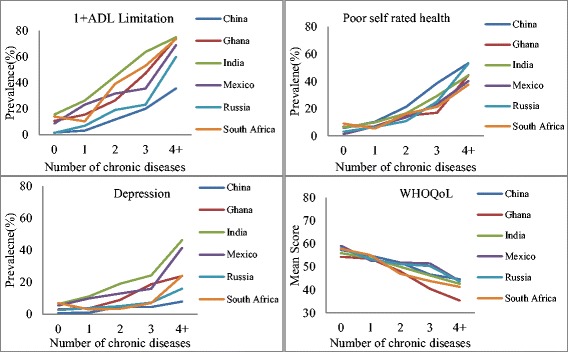
Prevalence of 1+ ADL limitations, poor self-rated health, and depression and mean quality of life scores, by count of diseases and country, WHO SAGE Wave 1 (2007/10)

Figure 2 shows the patterns of the four health outcomes, by number of chronic conditions and country. Across all figures, there is a consistent pattern of poor health outcomes with an increasing number of chronic conditions. There is, however, variation by country and domains. The ADL limitations increased sharply and consistently across all six countries with increasing number of diseases; the largest increase was observed in India and the lowest in South Africa. Compared to adults with one or two chronic diseases, the level of depression was substantially higher among adults with three or more chronic diseases. WHOQoL declined steadily (quality of life worsened) and prevalence of poor SRH increased with the number of conditions for all six countries.

### Association of multimorbidity with the four health outcome measures

The effects of multimorbidity on ADL, depression, SRH, and WHOQoL are presented in Table 4. The table shows both adjusted (for control variables) and unadjusted estimates for each of the health outcomes. The count of chronic diseases has a statistically significant negative effect on all four health outcomes. Each health outcome showed poorer results as the number of chronic diseases increased. For ADL limitations, depression and poor SRH, the adjusted and unadjusted odds were similar. Those with three chronic diseases were more than four times as likely to have 1+ ADL, depression, and poor SRH as adults with no diseases. Compared with adults with no disease, those with four or more diseases were almost seven times more likely to have 1+ ADL (OR = 7.21), depression (OR = 7.33), and poor SRH (OR = 7.38). The WHOQoL index was an average of eight points lower (adjusted β = −8.93) for adults with three chronic diseases compared to adults with no diseases.

**Table 4:** Multilevel logit model estimates for the effects of disease count on the four health outcome measures, WHO SAGE Wave 1 (2007/10)

Number of chronic diseases	1+ ADL		Depression		Poor self-rated health		Mean WHOQoL score	
	Unadjusted OR (95 % CI)	Adjusted OR (95 % CI)	Unadjusted OR (95 % CI)	Adjusted OR (95 % CI)	Unadjusted OR (95 % CI)	Adjusted OR (95 % CI)	Unadjusted beta (95 % CI)	Adjusted beta (95 % CI)
No disease^R^								
1 disease	2.07***	1.51***	1.77***	1.62***	1.90***	1.50***	−4.26***	−1.28***
	(1.93–2.22)	(1.38–1.65)	(1.57–2.01)	(1.42–1.84)	(1.74–2.08)	(1.35–1.65)	(−4.58 to –3.94)	(−1.59 to –0.98)
2 diseases	4.08***	2.47***	2.80***	2.44***	3.38***	2.25***	−7.38***	−3.11***
	(3.78–4.39)	(2.26–2.72)	(2.48–3.18)	(2.14–2.82)	(3.09–3.69)	(2.03–2.5)	(−7.73 to –7.03)	(−3.46 to –2.77)
3 diseases	7.28***	3.81***	4.74***	4.05***	6.33***	4.03***	−10.74***	−5.79***
	(6.66–7.92)	(3.42–4.26)	(4.12–5.46)	(3.47–4.75)	(5.71–6.98)	(3.59–4.5)	(−11.19 to –10.28)	(−6.23 to –5.35)
4+ diseases	15.18***	7.21***	8.75***	7.33***	12.3***	7.38***	−14.6***	−8.93***
	(13.58–16.83)	(6.33–8.17)	(7.53–10.12)	(6.24–8.61)	(10.99–13.71)	(6.43–8.38)	(−15.16 to –14.03)	(−9.48 to –8.38)
Random part								
Country	0.69	1.24	0.73	0.73	0.16	0.30	7.41	5.99
	(0.17–2.36)	(0.3–4.24)	(0.16–2.52)	(0.16–2.52)	(0.03–0.55)	(0.07–0.97)	(−1.93–16.76)	(−1.24–13.22)
Province/state	0.24	0.30	0.43	0.43	0.17	0.21	8.57	3.99
	(0.16–0.35)	(0.21–0.44)	(0.28–0.64)	(0.28–0.64)	(0.11–0.24)	(0.14–0.3)	(5.68–11.46)	(2.49–5.49)
Individual	na	na	na	na	na	na	163.83 (161.6–166.0)	120.68 (118.9–122.4)

* *P* <0.1, ** *P* <0.05, *** *P* <0.01. Adjusted ORs/beta coefficients are controlled for the effects of background characteristics and health risk factors. na, not applicable. Estimates are obtained through MCMC algorithm available in MLWin

Table 5 presents the regression analyses for the association of individual diseases and disease pairs on each of the subjective health outcomes. In Table 5, the results of Model 1 show the effects of individual chronic diseases on subjective health outcomes, after adjusting for the effects of other diseases and the control variables. Model 2 presents the main effects as well as the interactions of the chronic disease pairs. Results from Model 1 show that all diseases, except hypertension, had statistically significant negative effects on the subjective health outcomes. For example, adults with arthritis were more than two times as likely to have 1+ ADL and depression, and 1.8 times more likely to report poor SRH. The effect on WHOQoL was also negative; the mean score was three points lower among adults with arthritis. The other chronic diseases, including angina, lung diseases, low vision, diabetes, and stroke showed similar negative effects on subjective health outcomes.

**Table 5:** Multi-level regression estimates showing the effects of combinations of chronic diseases on the four health outcome measures, WHO SAGE Wave 1 (2007/10)

	1+ ADL		Depression		Poor self-rated health		WHOQoL score	
	OR (95 % CI)		OR (95 % CI)		OR (95 % CI)		Beta coefficient (95 % CI)	
	Model 1	Model 2	Model 1	Model 2	Model 1	Model 2	Model 1	Model 2
Main effects								
Angina	2.15***	2.74***	1.98***	2.89***	2.15***	2.66***	−3.49***	−3.79***
	(1.96–2.34)	(2.35–3.2)	(1.76–2.21)	(2.32–3.56)	(1.97–2.35)	(2.25–3.11)	(−3.87 to –3.09)	(−4.47 to –3.11)
Arthritis	2.15***	2.75***	2.06***	2.54***	1.84***	2.28***	−2.96***	−3.69***
	(1.99–2.32)	(2.43–3.12)	(1.85–2.28)	(2.13–3.03)	(1.7–1.99)	(1.98–2.61)	(−3.28 to –2.64)	(−4.24 to –3.13)
Asthma	1.50***	1.84***	1.71***	2.24***	1.57***	1.72***	−2.61***	−3.18***
	(1.31–1.72)	(1.4–2.39)	(1.45–2.00)	(1.58–3.07)	(1.35–1.79)	(1.25–2.29)	(−3.2 to –2.02)	(−4.35 to –1.99)
Chronic lung disease	2.02***	2.48***	2.14***	2.28***	2.00***	2.8***	−2.74***	−3.36***
	(1.8–2.25)	(2.03–3.04)	(1.86–2.44)	(1.73–2.98)	(1.79–2.22)	(2.23–3.42)	(−3.21 to –2.26)	(−4.35 to –1.99)
Diabetes	1.31***	1.38**	1.16**	1.51**	1.83***	2.29***	−2.26***	−3.4***
	(1.16–1.47)	(1.07–1.76)	(0.97–1.35)	(1.08–2.06)	(1.61–2.07)	(1.79–2.88)	(−2.77 to –1.74)	(−4.35 to –1.99)
Hypertension	1.04	1.05	0.98	1.09	1.05	1.02	0.01	0.02
	(0.97–1.11)	(0.95–1.16)	(0.88–1.08)	(0.93–1.26)	(0.98–1.13)	(0.92–1.15)	(−0.25–0.26)	(−0.32–0.38)
Low vision	1.28***	1.33***	1.23***	1.25**	1.19***	1.26***	−0.89***	−0.94***
	(1.19–1.36)	(1.19–1.47)	(1.11–1.35)	(1.07–1.45)	(1.11–1.28)	(1.11–1.43)	(−1.15 to –0.62)	(−1.15 to –0.62)
Stroke	2.40***	2.36***	2.21***	2.17***	2.22***	2.18***	−4.28***	−4.25***
	(1.99–2.86)	(1.97–2.81)	(1.75–2.73)	(1.73–2.7)	(1.86–2.62)	(1.83–2.57)	(−5.08 to –3.5)	(−5.04 to –3.47)
Two-way interactions								
Arthritis × Angina		0.78***		0.64***		0.79**		0.69**
		(0.65–0.92)		(0.51–0.81)		(0.67–0.93)		(−0.09–1.5)
Arthritis × Chronic lung disease		0.73**		0.84*		0.82**		1.17**
		(0.57–0.9)		(0.64–1.08)		(0.66–1.03)		(0.11–2.2)
Arthritis × Asthma		0.8*		0.83		0.73**		1.34**
		(0.59–1.05)		(0.59–1.14)		(0.54–0.95)		(0.05–2.64)
Arthritis × Low vision		0.82**		0.99		0.95		0.14
		(0.7–0.94)		(0.81–1.22)		(0.82–1.11)		(−0.5–0.78)
Arthritis × Diabetes		0.95		0.88		0.87		1.56**
		(0.73–1.21)		(0.61–1.23)		(0.67–1.1)		(0.39–2.73)
Angina × Chronic lung disease		0.86*		1.17		0.78**		0.72*
		(0.69–1.06)		(0.88–1.52)		(0.63–0.95)		(−0.32–1.75)
Angina × Asthma		0.81*		0.85		0.86		0.71
		(0.6–1.06)		(0.61–1.13)		(0.65–1.1)		(−0.56–1.99)
Angina × Low vision		0.99		1.05		0.90*		−0.43
		(0.83–1.16)		(0.84–1.28)		(0.75–1.05)		(−1.19–0.33)
Angina × Diabetes		0.92		0.79*		0.96		−0.03
		(0.69–1.22)		(0.54–1.11)		(0.73–1.24)		(−1.3–1.23)
Asthma × Low vision		1.04		1.06		1.00		−0.08
		(0.78–1.36)		(0.77–1.45)		(0.76–1.31)		(−1.25–1.15)
Asthma × Diabetes		1.31		1.32		0.65**		1.26
		(0.81–2.02)		(0.77–2.07)		(0.41–0.99)		(−0.79–3.32)
Hypertension × Arthritis		0.96		1.02		0.92		0.28
		(0.83–1.11)		(0.83–1.23)		(0.79–1.06)		(−0.36–0.92)
Hypertension × Angina		0.84**		0.63***		1.03		0.04
		(0.7–0.99)		(0.5–0.79)		(0.87–1.22)		(−0.71–0.78)
Hypertension × Lung disease		0.93		1.05		0.93		−0.10
		(0.75–1.17)		(0.8–1.36)		(0.76–1.15)		(−1.05–0.84)
Hypertension × Asthma		1.02		1.01		1.53***		−1.07**
		(0.77–1.32)		(0.72–1.37)		(1.16–1.99)		(−2.28–0.16)
Hypertension × Low vision		1.06		1.01		1.03		0.02
		(0.93–1.21)		(0.84–1.22)		(0.89–1.19)		(−0.49–0.55)
Hypertension × Diabetes		1.25**		1.11		1.12		−0.37
		(0.98–1.57)		(0.79–1.53)		(0.87–1.41)		(−1.4–0.64)
Low vision × Diabetes		0.74**		0.7**		0.85*		1.73***
		(0.57–0.92)		(0.5–0.95)		(0.66–1.06)		(0.71–2.73)
Chronic lung disease × Asthma		0.86		0.76**		0.90		0.54
		(0.65–1.12)		(0.54–1.02)		(0.68–1.17)		(−0.69–1.77)
Chronic lung disease × Low vision		1.10		0.99		0.92		−0.30
		(0.88–1.35)		(0.76–1.28)		(0.75–1.12)		(−1.26–0.64)
Chronic lung disease × Diabetes		1.05		1.00		0.67**		0.39
		(0.74–1.48)		(0.66–1.45)		(0.47–0.91)		(−1.23–1.97)
Random part								
Country	1.04	0.97	0.80	0.85	0.26	0.25	9.71	10.38
	(0.24–3.56)	(0.21–3.31)	(0.17–2.89)	(0.18–3.02)	(0.05–0.91)	(0.05–0.84)	(2.18–34.24)	(2.35–35.84)
Province	0.30	0.30	0.41	0.39	0.21	0.21	3.66	3.67
	(0.2–0.43)	(0.2–0.43)	(0.25–0.63)	(0.24–0.62)	(0.14–0.31)	(0.14–0.31)	(2.5–5.3)	(2.49–5.34)
Individual	Na	na	na	na	na	na	116.5 (114.7–118.4)	116.4 (114.5–118.2)

* *P* <0.1, ** *P* <0.05, *** *P* <0.01. All models control for background characteristics and health risk factors. na, not applicable. Estimates are obtained through MCMC algorithm available in MLWin; there are total 28 pairs of eight diseases, but we excluded seven interactions with stroke due to the small number of observations

Results of Model 2 reveal that the main effects on the different subjective health outcomes for all the conditions except hypertension were significant and negative. Subjective health measures were worse for adults with chronic diseases. The ‘main effects’ indicate the effect of each disease on a person suffering from none of the other conditions. The interaction estimates for the pairs of diseases showed a mixed pattern. While only a few of the interactions were significant, some of the disease pair interaction terms were positive, and some were negative. A positive interaction (odds ratio greater than one in logit models of 1+ ADL, depression, and poor SRH and negative coefficients in linear regressions for WHOQoL) shows that the combined effect of two diseases was more than the additive effect of each one of them individually; while a negative interaction (odds ratio less than one in logit models of 1+ ADL, depression, and poor SRH and positive coefficients in linear regressions for WHOQoL) indicates that the effect of the two diseases was less than the additive effect of each of them individually. The positive interactions show synergistic effects of the pair of diseases and the negative interactions show antagonistic effects.

Regression results for 1+ ADL showed that the interactions of nine disease pairs were statistically significant: hypertension-angina, hypertension-diabetes, arthritis-angina, arthritis-lung diseases, arthritis-asthma, arthritis-low vision, angina-chronic lung diseases, angina-asthma, and low vision-diabetes. Of these nine, the hypertension-diabetes was synergistic. For depression, the interactions of six disease pairs were statistically significant: hypertension-angina, arthritis-angina, arthritis-chronic lung diseases, angina-diabetes, chronic lung diseases-asthma, and low vision-diabetes; but no synergistic interactions emerged. For poor SRH, interactions with nine disease pairs were statistically significant: hypertension-asthma, arthritis-angina, arthritis-lung diseases, arthritis-asthma, angina-chronic lung disease, angina-low vision, chronic lung diseases-diabetes, asthma-diabetes, and low vision-diabetes. Of these significant interactions, only the hypertension-asthma pairing was synergistically associated with poor SRH. Similarly, for WHOQoL, interactions with seven disease pairs were significant: hypertension-asthma, arthritis-angina, arthritis-chronic lung disease, arthritis-asthma, arthritis-diabetes, angina-chronic lung diseases, and low vision-diabetes. The pairing of hypertension-asthma showed synergistic interactions with the WHOQoL results.

## Discussion

In this study, the prevalence and correlates of multimorbidity (encompassing eight chronic diseases – angina pectoris, arthritis, asthma, chronic lung disease, diabetes mellitus, hypertension, stroke, and low visual acuity) among adults in six LMICs was assessed. The impact of multimorbidity on four health outcome measures (ADL, depression, SRH, and quality of life) was examined.

The results show that more than half of the sample had at least one chronic disease and around one-fifth had multimorbidity. Among the six countries, Russia had the highest prevalence of multimorbidity (35 %) and China had the lowest (20 %). Ghana, India, Mexico, and South Africa had similar levels of multimorbidity, at around 22%. Further, it may be noted here that the high prevalence of measured hypertension in all six SAGE countries (ranging between 24 % in India to 51 % in South Africa) contributes substantially to the overall prevalence of multimorbidity. A comparative assessment of the multimorbidity with inclusion and exclusion of hypertension demonstrates that, with the exclusion of hypertension, the prevalence of multimorbidity is remarkably lower (21 % in Russia and 9 % in China) than that with the inclusion of hypertension in multimorbidity measure (Table 1 in Additional file 1).

The prevalence of multimorbidity was lower at higher levels of education in all six countries, demonstrating overall correlation of low SES with multimorbidity. Household wealth was negatively associated with multimorbidity for China and Russia, whereas the other four countries did not show a consistent pattern. The results of negative SES gradient of multimorbidity are consistent with the findings of previous studies [18, 21, 26, 36]. The SES gradient for multimorbidity was the sharpest for Russia, while the SES gradient was smallest for Ghana and India. The insignificant or inconsistent pattern of multimorbidity prevalence by household wealth in the lower income countries could be attributed to apparently contrasting socioeconomic patterns of NCD risk factors. Such patterns may arise on account of better access to health care and awareness about prevention and control of NCD risk factors among the wealthier stratum in high- and middle-income countries on one hand, while on the other hand, the wealthier stratum in lower income countries look to have higher levels of health risks like high BMI, high waist-hip ratio, cholesterol, and reduced physical activity [52–55]. Hosseinpoor et al. [56] have shown that the magnitude and direction of socioeconomic inequalities showed different patterns across risk factors such as sex and country income group. The adoption of risky health behaviors tends to transition from higher to lower socioeconomic groups as countries grow richer [57]. Analysis of cross-sectional correlates of multimorbidity suggests that multimorbidity is higher among older adults, women, and those with lower educational levels. These results are consistent with findings from several other studies [18, 36, 58, 59].

These six LMICs studied are home to a large proportion (42 %) of the world’s older population: a population at risk of the NCDs included in this study [60–63]. China faces a steep increase in chronic NCDs [64, 65]. India has to deal with an ongoing high burden from infectious and parasitic diseases as well as a rapidly rising burden from chronic diseases [66]. Mexico has seen a 52 % rise (from 23 % to 75 %) in the proportion of deaths from NCDs over the last 50 years [67], with NCDs now the leading cause of death and disease, representing 43 % of deaths and 51 % of disability-adjusted life years [68]. While Ghana has recognized the growing burden of chronic disease since the early 1990s, it is yet to implement a chronic disease policy or an integrated plan to deal with the issue [69]. In Russia, in the face of high adult mortality, expectancy began to decline in the mid-1960s and continued until the 2000s; such changes were mainly due to mortality from preventable causes, including chronic diseases and related risk factors and a lack of comprehensive disease prevention programs for NCDs [70–73]. South Africa is in the midst of a health transition characterized by a quadruple burden of communicable, non-communicable, and perinatal and maternal diseases, and interpersonal violence [74, 75]. The growing burden of NCDs requires concerted action from each of the governments in these countries.

Overall, the data reported herein indicate that multimorbidity has a significant impact on older adult physical and mental health outcomes in LMICs. The results confirm the negative effect of multimorbidity on quality of life, physical functioning, and mental health in LMICs, as has been previously demonstrated in HICs [26, 76–79]. Studies from HICs have further documented the synergistic effects of multimorbidity and socioeconomic deprivation [17, 78]; however, in contrast, no significant synergistic effects of number of diseases and socioeconomic deprivation were observed from this analysis (results are not presented).

The published literature suggests interactions between diseases should be considered to estimate contributions to health outcomes [80]; therefore, regression models were used in this study to estimate the effects of individual chronic diseases and disease pairs (interactions) on the four health outcome measures. The estimates revealed a significant and negative independent effect of each of the chronic diseases on ADL limitation, depression, poor SRH, and quality of life. The assessment of interaction effects of chronic disease pairs can help inform strategies for the prevention, control, and treatment of chronic diseases. Among disease pairs, the interaction of hypertension with most other NCDs was more than their additive effect on ADL limitation, SRH, and quality of life. No significant synergistic interaction was found for depression. These results contribute data from LMICs to the emerging evidence base on the nature of disease interactions in multimorbidity [26, 29, 76, 81]. Results also showed significant antagonistic interactions for all measures of health; interaction effects of two chronic diseases is equal to or lower than the combined effects of each of the individual diseases, rather than simple additive effects. These data possibly lend support to evidence that suggests that an individual’s level of functioning is reflected not by a simple sum of functioning across domains, but by the impact of diseases on the maximally affected domain and the number of domains affected [82].

The finding that multimorbidity is associated with adverse health outcomes has critical health care implications for people with multimorbidity in LMICs. Persons with multimorbidity need more inpatient and ambulatory care [83–85]. However, patients with multimorbidity also are at higher risk of iatrogenic disease and fragmentation of care because the treatment in such cases is often focused on one chronic condition [86]. Most clinical evidence and guidelines are created by individual disease and rarely account for multimorbidity [87, 88]. Given these results, the management of multimorbidity will surely become a considerable challenge for health systems worldwide [89] and requires an integrated people-centered service delivery approach with strengthening of primary health care systems [90]. The challenge is more pressing for LMICs, with health care systems often ill-equipped and largely focused on acute illnesses and maternal and child health care [5, 91].

Noting the potential bias introduced by disease prevalence derived from self-reported physician diagnosis [92–95], this study incorporated a number of alternate methods of estimating disease – using a mixture of self-reported diagnosis, validated symptom reporting-based diagnostic algorithms, and objective health measurements. This makes the findings that multimorbidity is also strongly associated with poor health outcomes in LMICs all the more striking and of major importance in public health and policy terms.

The findings from this study should be viewed in light of important limitations. First, we have used a count of chronic conditions as a measure of multimorbidity, which implies that each of the diseases has equivalent impact on an individual. In reality, the effects of multimorbidity on various domains of health are likely to depend on disease severity, the unique combination of diseases, and access to treatment and support. Second, we have modelled main effects of diseases with interaction terms between disease dyads (Table 5) to assess the effects of each disease pair on each of the four health outcomes. Here, we did not address the interaction of three and higher order interactions due to data limitations (insufficient observations). A third possible limitation relates to the measurement of hypertension in this study. The classification of hypertension based on an average of three measurements at the interval of 1 minute may have contributed to overestimation of hypertension prevalence compared to what may arise from measurement based on regular 24 h monitoring. Finally, the number of diseases included in this analysis was limited to those included in the SAGE study and, as such, may be missing some higher burden conditions, such as dementia and cancers, which could have resulted in an underestimation of the prevalence and impacts of multimorbidity [96]. However, a number of studies have analyzed multimorbidity using a smaller number of diseases, usually less than 10, due to data limitations in LMICs [97, 98]. Regardless, the prevalence found herein is striking and the reality likely to be even more confronting if all health conditions captured.

## Conclusions

The findings provide novel epidemiological evidence of the impact of multimorbidity on selected health outcome measures for six LMICs which have not previously been explored in such detail. Understandably, to date, LMICs have focused on infectious disease, malnutrition, and childhood health. However, these results indicate that there is a growing need to provide effective services for older adults to counter the impact of chronic multimorbidity on physical and mental health. In particular, the high prevalence of ADL limitations indicates the need for services for older adults. More research is required to assess the gaps in the community resources for providing services that maintain quality of life in the face of declining health.

## Additional file

Additional file 1:
**Symptoms and algorithms.** This file contains questions and corresponding algorithms to derive symptom-based prevalence of the chronic conditions assessed in this paper. The file also contains list of variables included for generating household wealth index.

## Abbreviations

ADL: Activities of daily living
HIC: High income countries
LMICs: Low- and middle-income countries
NCD: Non-communicable diseases
RRR: Relative risk ratio
SAGE: Study on Global Ageing and Health
SES: Socioeconomic status
SRH: Self-rated health
WHO: World Health Organization
WHOQoL: World Health Organizations’ Quality of Life instrument
