# Supplementary material for: The impact of multimorbidity on adult physical and mental health in low- and middle-income countries: what does the study on global ageing and adult health (SAGE) reveal?
Source: BMC Med. 2015 Aug 3;13:178. doi: 10.1186/s12916-015-0402-8 (PMC4524360; doi:10.1186/s12916-015-0402-8)
Supplement: Additional file 1: — Symptoms and algorithms. This file contains questions and corresponding algorithms to derive symptom-based prevalence of the chronic conditions assessed in this paper. The file also contains list of variables included for generating household wealth index. [file 12916_2015_402_MOESM1_ESM.docx]

Table1: Prevalence of multimorbidity with and without hypertension in SAGE countries, WHO-SAGE 2007/10.

| **Country** | **Multimorbidity (Without hypertension)** | **Multimorbidity (With hypertension)** | **Hypertension** |
| --- | --- | --- | --- |
|  |  |  |  |
| **China** | 9.05 | 20.34 | 42.28 |
| **Ghana** | 8.4 | 22.05 | 44.42 |
| **India** | 14.71 | 21.97 | 23.61 |
| **Mexico** | 12.05 | 22.06 | 29.52 |
| **Russian Federation** | 21.51 | 34.69 | 39.04 |
| **South Africa** | 8.85 | 22.46 | 51.16 |
| **Total** | 11.88 | 21.87 | 35.13 |

Table2: Symptoms and algorithms used to derive prevalence of individual chronic diseases in the SAGE Wave 1, 2007/10.

| **Disease name** | **Questions No** | **Question Text and algorithm to ascertain diseases** |
| --- | --- | --- |
| **Arthritis** | 1 | During the last 12 months, have you experienced, pain, aching, stiffness or swelling in or around the joints (like arms, hands, legs or feet) which were not related to an injury and lasted for more than a month? |
|  | 2 | During the last 12 months, have you experienced stiffness in the joint in the morning after getting up from bed, or after a long rest of the joint without movement? |
|  |  | If yes to question 2 |
|  | 3 | How long did this stiffness last? (1) less than 30 mins or 2) more than 30 mins |
|  | 4 | Did this stiffness go away after exercise or movement in the joint? 1.yes 2. No |
|  | **Algorithm** | **If response for questions 1 & 2 was yes and for 3 and 4 was first option then the respondent was said to have arthritis** |
| **Angina** | 1 | During the last 12 months, have you experienced any pain or discomfort in your chest when you walk uphill or hurry? |
|  | 2 | During the last 12 months, have you experienced any pain or discomfort in your chest when you walk at an ordinary pace on level ground? |
|  |  | If yes to question 2 |
|  | 3 | What do you do if you get the pain or discomfort when you are walking?(1 Stop or slow down 2 Carry on after taking a pain relieving medicine that dissolves in your mouth 3 Carry on walking) |
|  | 4 | If you stand still, what happens to the pain or discomfort? (1 Relieved 2 Not relieved) |
|  | 5 | Apart from these questions respondents were asked to identify the points of pain in the upper part of the body (excluding head) with help of a picture depicting upper parts of the body |
|  | **Algorithm** | **If the response to questions 1 & 2 was yes and for 3 & 4 it was first option & from the question 5 pain was in the left upper part of body the person was said to have angina** |
| **Lung diseases** | 1 | During the last 12 months, have you experienced any shortness of breath at rest? (while awake) |
|  | 2 | During the last 12 months, have you experienced any coughing or wheezing for ten minutes or more at a time? |
|  | 3 | During the last 12 months, have you experienced any coughing up sputum or phlegm for most days of the month for at least 3 months? |
|  | **Algorithm** | **a respondent was ascertained to have chronic lung disease if his response was yes to question 1 or yes to questions 2 and 3 both** |
| **Asthma** | 1 | During the last 12 months, have you experienced Attacks of wheezing or whistling breathing? |
|  | 2 | During the last 12 months, attack of wheezing that came on after you stopped exercising or some other physical activity? |
|  | 3 | During the last 12 months, a feeling of tightness in your chest? |
|  | 4 | During the last 12 months, have you woken up with a feeling of tightness in your chest in the morning or any other time? |
|  | 5 | During the last 12 months, have you had an attack of shortness of breath that came on without obvious cause when you were not exercising or doing some physical activity? |
|  | **Algorithm** | **A respondent was said to suffer from asthma if he responded yes question1 and yes to any of the subsequent questions (2-5).** |
| **Depression** | 1 | During the last 12 months, have you had a period lasting several days when you felt sad, empty or depressed? |
|  | 2 | During the last 12 months, have you had a period lasting several days when you lost interest in most things you usually enjoy such as personal relationships, work or hobbies/recreation? |
|  | 3 | During the last 12 months, have you had a period lasting several days when you have been feeling your energy decreased or that you are tired all the time? |
|  |  | If any of the above three questions are yes then following set of questions were asked |
|  | 4 | Was this period [of sadness/loss of interest/low energy] for more than 2 weeks? |
|  | 5 | Was this period [of sadness/loss of interest/low energy] most of the day, nearly every day? |
|  | 6 | During this period, did you lose your appetite? |
|  | 7 | Did you notice any slowing down in your thinking? |
|  | 8 | Did you notice any problems falling asleep? |
|  | 9 | Did you notice any problems waking up too early? |
|  | 10 | During this period, did you have any difficulties concentrating; for example, listening to others, working, watching TV, listening to the radio? |
|  | 11 | Did you notice any slowing down in your moving around? |
|  | 12 | During this period, did you feel anxious and worried most days? |
|  | 13 | During this period, were you so restless or jittery nearly every day that you paced up and down and couldn’t sit still? |
|  | 14 | During this period, did you feel negative about yourself or like you had lost confidence? |
|  | 15 | Did you frequently feel hopeless - that there was no way to improve things? |
|  | 16 | During this period, did your interest in sex decrease? |
|  | 17 | Did you think of death, or wish you were dead? |
|  | 18 | During this period, did you ever try to end your life? |
|  | **Algorithm** | **To ascertain the depression from this set of questions two set of variables were computed. First set was based on the questions1, 2, 3, 4, 5 and 16. From this set three variables were computed taking values 0 and 1: a) first variable takes value 1 if response to any of questions 1, 4, and 5 was yes. b) second variable takes value 1 if question 2 or 16 has response yes. c) the third variable takes value 1 if question 3 has response yes. The second set of variables was based on questions 6, 7, 8, 9, 10, 11, 12, 13, 14, 15, 17 and 18. From these questions seven variables were computed. a)first variable takes value 1 if response to questions 14 or 15 is yes. b) second variable takes value 1 if response to questions 12 or 13 is yes. c) third variable takes value 1 if questions 17 or 18 has response yes. d) fourth variable takes value 1 if questions 7 or 10 has response yes. e) fifth variable takes value 1 if response to questions 11 is yes. f) sixth variable takes value 1 if response to questions 8 or 9 is yes. g) seventh variable takes value 1 if the response to question6 is yes. These newly created variable from the respective sets were added to obtain two new variables : first consisting sum of first set of variables (maximum value 3) and second consisting sum of second set of variables (maximum value 7). Based on these two variables, a respondent is said to suffer from depression if he has value for the first variable to be 2-plus and the value for second variable to be 4-plus.** |

Table3: List of items used for calculation of household wealth, WHO-SAGE India, 2007

| **Question No.** | **Item details** |
| --- | --- |
| q0700 | Can you please tell me how many rooms there are in your home? |
| q0701 | How many chairs are there in your home? |
| q0702 | How many tables are there in your home? |
| q0703 | How many cars are there in your household? |
| q0704 | Does your home have electricity? |
| q0705 | Does anyone in your household have a bicycle? |
| q0706 | Does anyone in your household have a clock? |
| q0707 | Does anyone in your household have a bucket? |
| q0708 | Does anyone in your household have a washing machine for clothes? |
| q0709 | Does anyone in your household have a refrigerator? |
| q0710 | Does anyone in your household have a refrigerator? |
| q0711 | Does anyone in your household have a fixed line telephone? |
| q0712 | Does anyone in your household have a mobile/cellular telephone? |
| q0713 | Does anyone in your household have a television? |
| q0714 | Does anyone in your household have a computer? |
| q0715 | Does anyone in your household have moped/scooter/motorcycle? |
| q0716 | Does anyone in your household have live-stock (cattle only)? |
| q0717 | Does anyone in your household have sewing machine? |
| q0718 | Does anyone in your household have radio/transistor/tape recorder? |
| q0719 | Does anyone in your household have bullock cart? |
